# Impact of Visual Field Testing on Intraocular Pressure Change Trends in Healthy People and Glaucoma Patients

**DOI:** 10.1155/2020/7936205

**Published:** 2020-07-04

**Authors:** Mengwei Li, Bingxin Zheng, Qi Wang, Xinghuai Sun

**Affiliations:** ^1^Department of Ophthalmology and Visual Science, Eye, Ear, Nose and Throat Hospital, Shanghai Medical College of Fudan University, Shanghai, China; ^2^NHC Key Laboratory of Myopia (Fudan University), Key Laboratory of Myopia, Chinese Academy of Medical Sciences, Shanghai Key Laboratory of Visual Impairment and Restoration (Fudan University), Shanghai, China; ^3^Department of Nursing, Eye, Ear, Nose and Throat Hospital, Shanghai Medical College of Fudan University, Shanghai, China; ^4^State Key Laboratory of Medical Neurobiology, Institutes of Brain Science and Collaborative Innovation Center for Brain Science, Fudan University, Shanghai, China

## Abstract

**Purpose:**

To compare the impact of visual field (VF) testing on intraocular pressure (IOP) change trends between healthy subjects and glaucoma patients.

**Methods:**

We recruited healthy volunteer subjects who did not have previous ocular diseases and open-angle glaucoma patients who were medically controlled well. IOP in both eyes of each participant was measured by using a noncontact tonometer at five time points: before, immediately after (0 minute), and 10, 30, and 60 minutes after the standard automated perimetry. Repeated measures ANOVA was used to analyze the effect of VF testing on IOP change trends in healthy and glaucoma eyes.

**Results:**

Forty healthy subjects (80 eyes) and 31 open-angle glaucoma patients (62 eyes) were included for the study. The baseline IOP of healthy and glaucoma eyes was 16.11 ± 3.01 mmHg and 15.78 ± 3.57 mmHg, respectively. After the VF testing, the IOP in healthy eyes was decreased by 1.5% at 0 minute, 6.5% at 10 minutes (*P* < 0.001), 6.6% at 30 minutes (*P* < 0.001), and 7.0% at 1 hour (*P* < 0.001), indicating that this reduction was sustained for at least 1 hour. However, the IOP in glaucoma eyes was increased by 12.7% at 0 minute (*P* < 0.001) and, then, returned towards initial values 1 hour after the VF testing.

**Conclusions:**

IOP change trends after VF field testing between healthy subjects and glaucoma patients were quite different. VF testing led to a mild and relatively sustained IOP decrease in healthy subjects, whereas IOP in open-angle glaucoma patients tended to significantly increase immediately after VF testing and, then, returned to pretest values after 1 hour. These findings indicate that the factors of VF testing should be considered in the clinical IOP measurements.

## 1. Introduction

As elevated intraocular pressure (IOP) is a major risk factor in the development of glaucoma that can severely threaten our visual function [1–3], accurate IOP measurements are very essential for daily management of this disease [4]. However, IOP is a highly variable and dynamic parameter and is affected by numerous factors, for example, measurement factors (such as tonometer and examiner), ocular factors (such as corneal thickness, corneal hysteresis, and dehydration), and individual factors (such as accommodation, circadian cycle, body position, mental stress, and blood pressure) [5, 6]. It is, therefore, of paramount significance for ophthalmologists to know about IOP variations and their influences in the clinical treatment and follow-up of glaucoma patients.

Visual field (VF) examination is another key component of the clinical assessment of glaucoma or glaucoma suspect, often along with IOP measurements during the same visit [1, 7]. In our clinical practice, IOP is sometimes measured after automated static perimetry. Whether VF testing affects IOP levels still remains controversial. Some studies showed that IOP increased in eyes with primary open-angle glaucoma (POAG) after automated VF testing [8, 9]. However, contradictory findings have also been reported, with the conclusion that VF examination did not significantly influence IOP in POAG patients [10–12]. We noted that, in all aforementioned studies, IOPs were measured only at two or three time points. As physiological variations exist in IOP [5], such few measuring time points could be insufficient to reflect real IOP change trends after VF testing and might be the reason of contradictory results in previous studies.

Thus, the purpose of this study was to measure IOP at multiple time points after automated VF examination so as to determine the impact of VF testing on IOP change trends in healthy subjects and glaucoma patients.

## 2. Materials and Methods

### 2.1. Participants

This was a prospective cross-sectional clinical study that was conducted between Dec. 2017 and Jun. 2019 in the Glaucoma Clinic of Eye and ENT Hospital of Fudan University. We recruited healthy volunteer subjects who did not have prior ocular diseases and open-angle glaucoma patients who were medically controlled well. The procedures of our research were approved by the human subjects review committee of Eye and ENT Hospital of Fudan University, China, and conformed to the tenets of the declaration of Helsinki. All participants provided written informed consent before participation.

The inclusion criteria of glaucoma patients were as follows: (1) open-angle glaucomas including POAG (untreated IOP >21 mmHg) and normal tension glaucoma (NTG) (untreated IOP ≤21 mmHg over multiple measurements during follow-up) with evidence of characteristic glaucomatous optic nerve excavation corresponding to the location of the VF defects; (2) well-controlled and stable IOP <21 mmHg under medical treatment (prostaglandins, *β*-blockers, *α*2-agonists, and carbonic anhydrase inhibitors) during two years of follow-up before the study; (3) nonprogressive optic disk damage and VF defect assessed by a glaucoma specialist (X.S.) for more than two years of follow-up; and (4) aged 20–70 years. The exclusion criteria were as follows: (1) history of any intraocular surgery including laser therapy; (2) history of ocular injury or other intraocular diseases that affect the VF testing; (3) any corneal disease that prevented reliable IOP measurements; (4) patients who were receiving pilocarpine that affected the pupil diameter or accommodation and who were taking long acting medications; (5) high myopia with a spherical equivalent (SE) <−6.0 diopter (D) or an axial length ≥26.5 mm; and (6) the best corrected visual acuity was below 20/200 in at least one eye.

Healthy subjects selected for inclusion were those aged 20–70 years who had no ocular disease (besides mild cataract) and could cooperate with the VF testing. Subjects who had high myopia (SE < −6.0D or an axial length ≥26.5 mm) in any eye and were taking long acting medications were excluded.

### 2.2. Examinations

Each healthy subject and glaucoma patient participated in this study and underwent the same procedure in the morning (8:30 a.m. to 11:30 a.m). The IOP measurements in both eyes (the right eye first, and then, the left eye) of the participants were performed with a noncontact tonometer (TX-20, Canon, Japan) by a single experienced operator (B.Z.). These IOPs were measured in five time points: immediately before (baseline), immediately after (0 minute), 10 minutes after, 30 minutes after, and 60 minutes after the VF testing. A total of three consecutive sets of IOPs in each eye were obtained at each time point, and the mean of these three IOPs was used as the final value of each time point for the statistical analyses. The VF testing we performed was a standard automated perimetry (Humphrey Field Analyzer; 750 I series; Carl Zeiss Meditec, Dublin, California, USA) with the central 30-2 Swedish interactive threshold algorithm (SITA) program. During the testing, an appropriate near-prescription lens was added as needed, and the fellow eye was patched with gauze. We examined the right eye first and, then, the left eye in the VF testing, which took about 4–9 minutes for each eye. Finally, central corneal thickness (CCT) was measured with a low-coherence interferometer (LenStar 900; Haag-Streit, Koeniz, Switzerland).

### 2.3. Statistical Analysis

All statistical analyses were performed with IBM SPSS software version 20.0 (SPSS, Inc., Chicago, IL, USA). The independent *t* test, Mann–Whitney *U* test, and Chi-square test were used to evaluate between-group differences with respect to the demographic characteristics. Within-group changes of IOP over time were analyzed by one-way repeated measures ANOVA, followed by Bonferroni correction for pairwise comparisons. Between-group differences of IOP trends were compared by two-way repeated measures ANOVA. The time-by-group interaction was tested first. If significant, between-group differences at each time point were implemented by the independent *t* test. If not significant, the main effect (group) was tested next. The significance level was set at 0.05 (two-tailed).

## 3. Results

A total of 71 participants (142 eyes) including 40 healthy subjects (80 eyes) and 31 open-angle glaucoma (19 POAG and 12 NTG) patients (62 eyes) were included for the study. Mean ages (±standard deviation (SD)) of healthy subjects and glaucoma patients were 43.1 ± 12.6 years and 45.0 ± 14.6 years, respectively. There were 19 males and 21 females in healthy subjects and 21 males and 10 females in glaucoma patients. The between-group differences with respect to age, gender, and other demographic values such as central corneal thickness (CCT) showed no statistical significance (all *P* > 0.05). Nevertheless, the absolute value of mean defect (MD) of Humphrey VF and the VF testing time in glaucoma eyes was significantly greater and longer, respectively, than that in healthy eyes (both *P* < 0.001) (Table 1).

**Figure 1:**
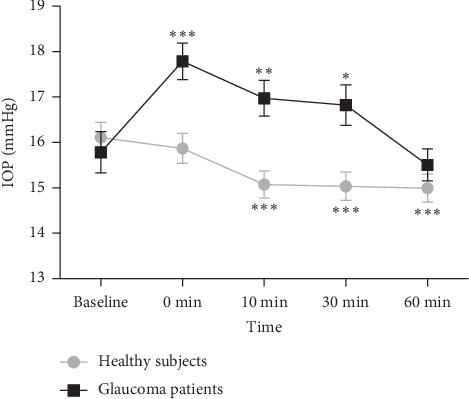
Intraocular pressure (IOP) fluctuation before (baseline) and after (0, 10, 30, and 60 minutes) visual field testing in healthy subjects and glaucoma patients. ^*∗∗∗*^*P* < 0.001, ^*∗∗*^*P* < 0.01, and ^*∗*^*P* < 0.05 compared with baseline (one-way repeated-measures ANOVA followed by Bonferroni correction). Error bar denotes SEM.

The baseline IOP values were similar between healthy eyes and glaucoma eyes (*P*=0.560). Two-way repeated measures ANOVA analysis revealed that a significant time-by-group interaction on IOP (*P* < 0.001), indicating the IOP change trends between groups were different. One-way repeated measures ANOVA analyses disclosed a significant effect of time on IOP in both groups (both *P* < 0.001). After the VF testing was performed, IOP levels of healthy eyes were found to be decreased by 1.5% at 0 minute, 6.5% at 10 minutes (*P* < 0.001), 6.6% at 30 minutes (*P* < 0.001), and 7.0% at 1 hour (*P* < 0.001), indicating that healthy people experienced a slight and relatively sustained decline in IOP after the VF examination in our 1 hour of observation. However, IOP levels of glaucoma eyes were observed to be increased by 12.7% at 0 minute (*P* < 0.001) and, then, returned towards initial values 1 hour after the VF testing (Table 2, Figure 1).

**Figure 2:**
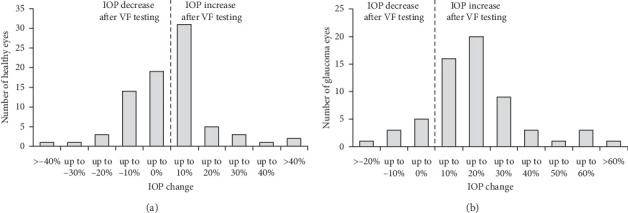
Distribution of intraocular pressure (IOP) change immediately after visual field (VF) testing in healthy eyes (a) and glaucoma eyes (b). IOP change is defined as a percent change in IOP measured immediately (0 minute) after the VF testing compared with the IOP measured before VF testing (baseline). 13.8% and 7.5% of the healthy eyes experienced >10% and >20% IOP increase, whereas 59.7% and 27.4% of the glaucoma eyes experienced >10% and >20% IOP increase.

On analyzing the distribution of IOP change immediately (0 minute) after VF testing in both groups, we calculated IOP change as the percent change of the IOP measured immediately after the VF testing compared with the baseline IOP. We found that 13.8% and 7.5% of the healthy eyes experienced more than 10% and more than 20% IOP increase, whereas 59.7% and 27.4% of the glaucoma eyes experienced more than 10% and more than 20% IOP increase immediately after the VF testing (Figure 2).

## 4. Discussion

Previous researches about the impact of VF testing on IOP have yielded inconsistent and conflicting results. Recupero et al. [8] found that VF testing led to a transient IOP increase of more than 2 mmHg in 44.7% of POAG patients, but no IOP change in healthy subjects. Similarly, Ni et al. [9] reported that more than 20% increase of IOP was obtained after VF testing compared with IOPs from the previous and next visits without VF testing in 22.9% of POAG patients. On the contrary, Rebolleda et al. [10] found that IOP did not vary significantly immediately after VF testing compared with 1 hour later in 27 POAG patients. Martin [11] also determined that there were no significant differences of IOP values between immediately before and immediately after routine VF testing in 40 treated glaucoma patients and 21 untreated ocular hypertension or suspected glaucoma. Meanwhile, Sawada et al. [12] concluded that VF testing did not lead to an increase of IOP in the majority of glaucoma eyes.

Several factors should be considered in these contradictory results: first, the IOP measurements in all the aforementioned studies only included two or three time points, making it hard to gain the real IOP change trends due to physiological variations or measurement error of IOP. Second, only one study compared the effect of VF testing on the IOP between glaucoma patients and healthy subjects in the same condition. Therefore, to date, the impact of VF testing on IOP change trends in both healthy people and glaucoma patients still remains unclear. In the present study, to analyze IOP change trends better, we measured IOP at five time points: before, immediately after, and 10 minutes, 30 minutes, and 1 hour after the VF testing. The magnitude of IOP change immediately after VF testing shows that nearly 60% of the glaucoma eyes had more than 10% IOP increase, while only about 13% of the healthy eyes had more than 10% IOP increase, indicating that the IOP responses to VF testing between these two population were different. IOP change trend analyses further reveal that, in the same circumstance, VF testing led to a mild decrease of IOP in healthy subjects as a whole, whereas IOP tended to significantly increase immediately after VF testing and, then, returned to the initial values after 1 hour in open-angle glaucoma patients.

When focusing on a fixation target in the VF examination, the eye uses the accommodative mechanism by changing the shape of its crystalline lens to maintain a clear retinal image [13]. It is well established that during accommodation, contraction of ciliary body muscles leads to mechanical traction on the scleral spur and trabecular meshwork, thus opening up the pores in the trabecular meshwork and increasing the aqueous outflow [14]. There has been mounting evidence that accommodation with lenses or near work causes a decrease of IOP in healthy subjects [15–18]. A recent research not only demonstrated that near work reduced IOP by about 1.87 mmHg in healthy emmetropes but also first revealed that this effect was sustained for at least 20 minutes after discontinuing near work [18]. In the present study, we found that VF testing reduced the IOP by about 1.12 mmHg (7.0%) in healthy subjects, and this reduction was sustained for at least 1 hour following VF testing, indicating that this hypotensive effect was not instantaneous, but could last for a period of time. Our results disagree with the previous study in which VF testing led to no IOP change in healthy subjects [8]. The discrepant finding may be due to only two IOP measuring time points (before and after VF testing) and small sample size (13 subjects) in that study, which could be insufficient to reflect the real IOP change trends. Our findings of VF testing-induced IOP changes in healthy subjects can be explained by the accommodative factor. Whether this hypotensive effect will continue after 1 hour needs further investigation.

Since accommodation can reduce IOP in healthy people, it is necessary to know about why IOP in glaucoma patients increased first and then returned to the initial values after VF testing. Actually, during accommodation, the state of contraction of ciliary muscle can not only increase the trabecular outflow by opening up the trabecular meshwork as mentioned above but also decrease the uveoscleral outflow by reducing the size of spaces between the ciliary bundles as well [14, 19, 20]. It is well known that increased resistance to trabecular outflow is the hallmark in the pathophysiology of open-angle glaucoma [21]. Therefore, we speculate that, during accommodation, the effect of increased trabecular outflow is larger than the effect of decreased uveoscleral outflow in healthy people; however, the decrease of the uveoscleral outflow might play a more important role than the effect of trabecular outflow due to increased resistance within the conventional outflow pathway in glaucoma patients. This could explain why the hypotensive effect appeared in healthy eyes, while the hypertensive effect happened to glaucoma eyes after VF testing in our study. In addition, it is also possible that glaucoma patients are more anxious than healthy subjects during VF testing. A large investigation shows that there is a statistical association between glaucoma and each of anxiety and depression [22]. Such psychological changes might stimulate sympathetic nerves and make norepinephrine release into the aqueous humor, leading to transiently elevate IOP in glaucoma eyes [23]. In the present study, we found that the average testing time of VF testing in glaucoma patients was longer than that in healthy subjects, which might lead to a higher level of anxiety in these patients. Whether the hypertensive effect after VF testing in glaucoma eyes is mainly based on the accommodative effect or psychological factors needs futher research.

There are some limitations to this study that we acknowledge. First, our sample size was relatively small though our results were statistically significant. Second, IOP was measured by a noncontact tonometer at each time point. Since IOP needed measure for several times in the same individual, it was impossible to use Goldmann applanation tonometry so as to avoid corneal injury. However, noncontact tonometry has been demonstrated to have similar accuracy to the Goldmann applanation tonometry [24, 25].

In conclusion, our results show that the IOP change trends after VF field testing in healthy subjects and glaucoma patients were quite different. VF testing led to a mild decrease of IOP in healthy subjects, and this reduction was sustained for at least 1 hour. However, IOP tended to significantly increase immediately after VF testing and then returned to pretest values after 1 hour in open-angle glaucoma patients. These findings indicate that the factors of VF testing should be considered in the clinical IOP measurements in healthy people and glaucoma patients.

## Figures and Tables

**Table 1:** Demographic characteristics of healthy subjects and glaucoma patients.

	Healthy subjects	Glaucoma patients	*P* value
Number	40	31	—
Age (years)	43.1 ± 12.6	45.0 ± 14.6	0.547^*∗*^
Gender (male/female)	19/21	21/10	0.088^†^
CCT (*μ*m)	536.2 ± 22.2	536.6 ± 20.8	0.930^*∗*^
MD (dB)	−1.75 (−3.15, −0.85)	−6.45 (−12.70, −3.41)	**<0.001** ^‡^
VF testing time (min)	5.60 ± 0.86	6.78 ± 1.13	**<0.001** ^*∗*^

Continuous values are described as mean ± standard deviation except that MD is presented as median (interquartile range). *P* values <0.05 are in bold. MD: mean defect; CCT: central corneal thickness; VF: visual field. ^*∗*^Independent *t*-test. ^†^Chi-square test. ^‡^Mann–Whitney *U* test.

**Table 2:** Comparison of IOP variations between healthy subjects and glaucoma patients before (baseline) and after (0, 10, 30, and 60 minutes) visual field testing.

IOP (mmHg)	Healthy subjects group (*n* = 80 eyes)	Glaucoma patients group (*n* = 62 eyes)	Between-group *P* value
Baseline	16.11 ± 3.01	15.78 ± 3.57	0.560
0 min	15.87 ± 2.94	17.79 ± 3.18	**<0.001**
10 min	15.07 ± 2.70	16.97 ± 3.08	**<0.001**
30 min	15.04 ± 2.79	16.82 ± 3.51	**0.001**
60 min	14.99 ± 2.76	15.55 ± 2.77	0.277

All values are described as mean ± standard. *P* Values <0.05 are in bold. IOP: intraocular pressure.

## Data Availability

The data used to support the findings of this study are available from the corresponding author upon request.
